# Mechanical Properties and Microstructure of TIG and ATIG Welded 316L Austenitic Stainless Steel with Multi-Components Flux Optimization Using Mixing Design Method and Particle Swarm Optimization (PSO)

**DOI:** 10.3390/ma14237139

**Published:** 2021-11-24

**Authors:** Abdeljlil Chihaoui Hedhibi, Kamel Touileb, Rachid Djoudjou, Abousoufiane Ouis, Hussein Alrobei, Mohamed M. Z. Ahmed

**Affiliations:** 1 Department of Mechanical Engineering, National Engineering School of Tunis (ENIT), El-Manar University, P.O. Box 37, Belvedere Tunis, Tunis 1002, Tunisia; a.hedhibi@psau.edu.sa; 2 Department of Mechanical Engineering, College of Engineering in Al-Kharj, Prince Sattam bin Abdulaziz University, P.O. Box 655, Al-Kharj 16273, Saudi Arabia; k.touileb@psau.edu.sa (K.T.); a.ouis@psau.edu.sa (A.O.); h.alrobei@psau.edu.sa (H.A.); 3 Department of Metallurgical and Materials Engineering, Faculty of Petroleum and Mining Engineering, Suez University, Suez 43512, Egypt

**Keywords:** ATIG welding, mixing design method, particle swarm optimization (PSO), pseudo-ternary flux, 316L SS, mathematical modeling, mechanical properties, microstructure

## Abstract

In this study, the effects of pseudo-ternary oxides on mechanical properties and microstructure of 316L stainless steel tungsten inert gas (TIG) and activating tungsten inert gas (ATIG) welded joints were investigated. The novelty in this work is introducing a metaheuristic technique called the particle swarm optimization (PSO) method to develop a mathematical model of the ultimate tensile strength (UTS) in terms of proportions of oxides flux. A constrained optimization algorithm available in Matlab 2020 optimization toolbox is used to find the optimal percentages of the selected powders that provide the maximum UTS. The study indicates that the optimal composition of flux was: 32% Cr_2_O_3_, 43% ZrO_2_, 8% Si_2_O, and 17% CaF_2_. The UTS was 571 MPa for conventional TIG weld and rose to 600 MPa for the optimal ATIG flux. The obtained result of hardness for the optimal ATIG was 176 HV against 175 HV for conventional TIG weld. The energy absorbed in the weld zone during the impact test was 267 J/cm^2^ for the optimal ATIG weld and slightly higher than that of conventional TIG weld 256 J/cm^2^. Fracture surface examined by scanning electron microscope (SEM) shows ductile fracture for ATIG weld with small and multiple dimples in comparison for TIG weld. Moreover, the depth of optimized flux is greater than that of TIG weld by two times. The ratio D/W was improved by 3.13 times. Energy dispersive spectroscopy (EDS) analysis shows traces of the sulfur element in the TIG weld zone.

## 1. Introduction

Austenitic stainless steels are the most common stainless steel. They are used in many fields as oil, shipbuilding, machinery, and marine applications. They are characterized by good strength, high toughness, and excellent corrosion resistance. Fusion welding is the most popular method to join workpieces in many industrial applications.

Tungsten Inert Gas (TIG) welding is a widespread process in industries, but a limited thickness can be joined in a single pass with this process. TIG welding is also very sensitive to the chemical composition of the base metal. Moreover, materials thickness greater than 3 mm requires multiple passes to achieve full penetration weld; therefore, the productivity of the process is reduced. The activated tungsten inert gas (ATIG) technic is a variant of conventional TIG welding. In ATIG welding, a thin layer of flux is deposited on the workpiece before the welding operation. ATIG offers the possibility of increasing the penetration depth using the same equipment and parameters as conventional TIG. Comparatively to TIG, ATIG has many advantages. It eliminates the need for edge preparation, increases the penetration depth, and reduces the number of weld pass [1,2]. Three mechanisms have been proposed; the increase in ATIG weld penetration can be attributed to the reversal of Marangoni convection [3,4]. The second mechanism is the arc constriction proposed by D. S. Howse et al. [5,6]. The third mechanism, proposed by Sire, consists of a constriction of arc by using a flux characterized by high melting point and high electrical resistivity [7,8]. In recent years, considerable works were carried out using Taguchi method design to optimize TIG welding process parameters to improve both depth and weld aspects [9,10]. Some studies were oriented to study the influence of welding parameters on enhancing mechanical properties [11,12]. Other works were dedicated to optimizing the composition of paste to improve the depth and weld aspects [13,14] using the mixing method. Recently, other methods like genetic algorithms, simulated annealing, particle swarm optimization [15], etc., have been used to optimize solution in many industries [16,17]. In this work, the tensile strength of weld joints is optimized as an output parameter by varying the input parameters that are the combinations of oxides powder without varying the welding parameters.

The objective of this work is to elaborate an appropriate composition of flux to improve mechanical properties of the full penetrated 316L stainless steel ATIG weld. Mixing method simplex design degree four was combined with particle swarm optimization (PSO) method to optimize combination powders. The developed mathematical model and optimization methodology can help researchers and engineers working in developing fluxes for weld improvements. This study is a contribution to expanding research dedicated to ATIG welding of 316L.

## 2. Materials and Methods

### 2.1. Material

The material used in this study is the austenitic stainless steel grade 316L. The chemical composition is shown in Table 1. Rectangular pieces of 6 mm thickness were cut from the received plate to perform welding of 20 cm line.

### 2.2. TIG-ATIG Welding Platform

**Figure 1:**
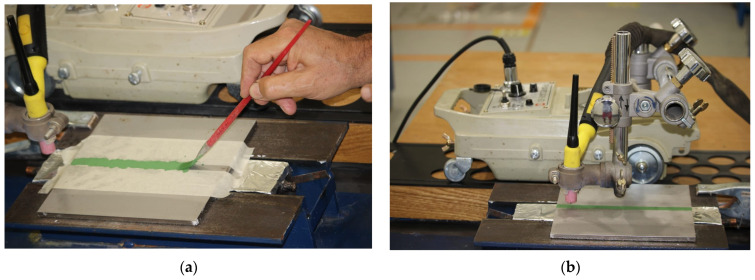
The deposition of flux on the workpiece (**a**), motorized carriage (**b**).

First of all, the plates were cleaned with acetone. The oxide powders were dried in the furnace for 1 h at 180 °C to eliminate the humidity. Then, a thin layer of a mixed powder with methanol was applied using a brush to the surface subject to the welding as shown in Figure 1a. The mean coating density of flux was about 4–5 mg/cm^2^.

The tungsten inert gas welding machine was used. The electrode used has a diameter of 3.2 mm and the torch was mounted on a motorized carriage as shown in Figure 1b. The experimental parameters selected for welding are presented in Table 2.

**Figure 2:**
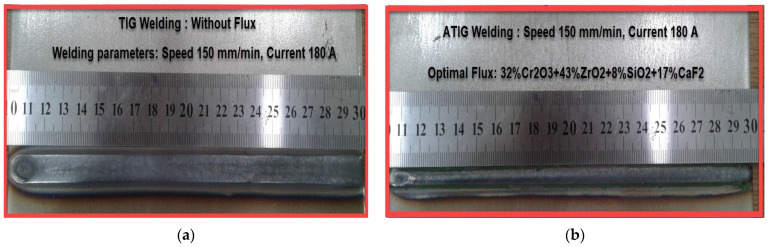
The external weld line appearance for TIG (**a**) and ATIG (**b**) weld lines.

Figure 2a shows a good external aspect for TIG weld line, also surface ATIG weld line in Figure 2b is free of slugs, which indicates that the optimal flux is well consumed with small traces of residue.

**Figure 3:**
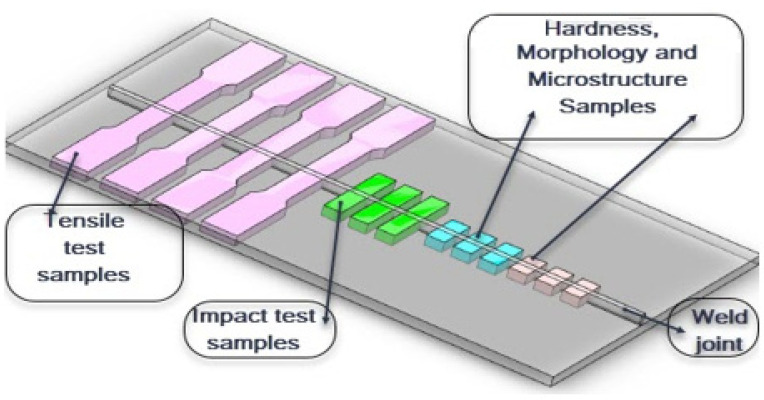
Schematic drawing showing test specimens.

Pieces were cut from the welded plates for mechanical testing, weld morphology, and microstructure study according to the schematic drawing shown in Figure 3. The cutting machine used is Hydro-Jet Eco 0515 SL-Micro waterjet Cutting (KNUTH Germany).

### 2.3. Tensile Test

Design of experiment and mathematical modelling will be applied to the tensile strength property. Optimal flux, which will give the maximum UTS resulting from the tensile test, is used for the rest of the study.

The tensile tests were performed with a computer control electrohydraulic servo universal testing machine model WAW-300E (Jinan testing equipment IE, Jinan, China), at a test rate of 0.5 mm/min, at 0.5 kN/s load rate, and at low strain rate of 1.6 × 10^−4^ s^−1^.

### 2.4. Weld Bead Aspect

The cross-sections of the weld beads for ATIG with optimal flux and conventional TIG were photographed using an optical microscope CAROLINA (CAROLINA, Burlington, NJ, USA). The morphology of the welds were checked using Motic Images plus version 2.0 software integrated with an optical microscope.

### 2.5. Microstructure Assessment

The microstructural characterization of the fusion zone of both TIG and ATIG welding has been analyzed. Micrographs were taken on JEOL JSM-7600F scanning electronic microscope, (SEM). The areas image processing software from Microvision Instruments (Microvision Instruments, Imager M2.m, Paris, France) was used to measure the ferrite volume proportions.

### 2.6. Hardness Test

**Figure 4:**
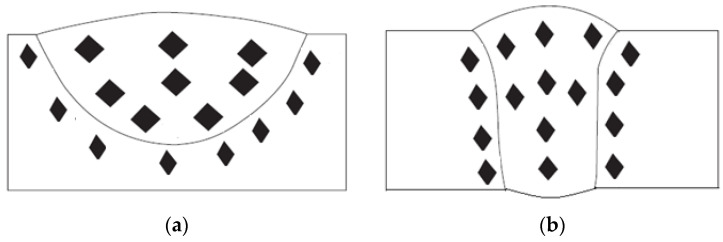
Hardness test locations in the FZ and the HAZ of TIG (**a**) and ATIG (**b**) [13].

Vickers hardness tests were performed by a digital hardness tester model HVS-50 (SCTMC, Shanghai, China) with a standard load of 98 N. Eight indentations in the weld bead on fusion zone (FZ) and at the heat-affected zone (HAZ) were performed on each sample, with about 0.5 mm between two indentations, as shown in Figure 4.

### 2.7. Impact Test

**Figure 5:**
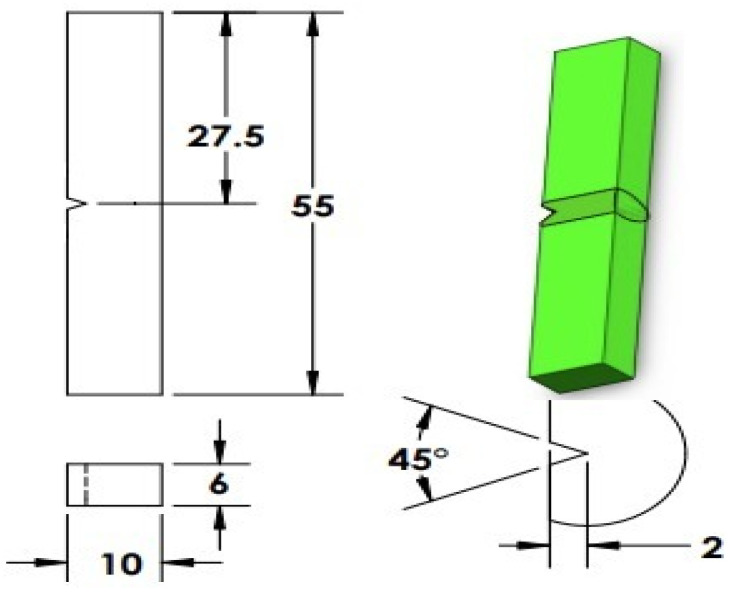
Sample for impact test “V” notch in the fusion zone (FZ) of TIG and ATIG Welded.

Impact testing was performed on three samples for TIG and three samples for ATIG weld only in the fusion zone (FZ) with the Charpy “V” notch impact testing machine model JBS-500 (Jinan testing equipment IE, Jinan, China), specimens are shown in Figure 5.

### 2.8. Design of Experiment Methodology

To get the maximum amount of useful information with the least amount of experimentation, the design of experiments (DOE) has been used; mixing method in Minitab 17 software was applied. First, to compare the effect of the mono-flux oxides on UTS of welds, thirteen oxides (SiO_2_, TiO_2_, Fe_2_O_3_, MnO_2_, Cr_2_O_3,_ ZrO_2_, CaO, Mn_2_O_3_, V_2_O_5_, MoO_3_, SrO, Co_2_O_3,_ and MgO) were tested. Among these thirteen oxides, three oxides Cr_2_O_3_, TiO_2_, and ZrO_2_ that gave the best UTS were selected to be used in the mixing design method. Based on the simplex lattice degree four designs, nineteen combinations from the selected oxides have been prepared. For each combination, the three selected oxides Cr_2_O_3_, TiO_2_, and ZrO_2_ vary and 25% of (8% SiO_2_ + 17% CaF_2_) was added and kept fixed to get a pseudo ternary combination. Particle swarm optimization (PSO) method is used to establish an equation relating UTS to the proportions of the selected oxides. Finally, Matlab R2020 is carried out to obtain the optimal combination of oxides, hence, to maximize the UTS of weld through a constrained optimization algorithm.

Silica (SiO_2_) has been added because it increases the current carrying capacity. Consequently, silicate (SiO_2_) increases arc voltage and depth penetration of the weld bead [18]. On the other hand, the addition of calcium fluoride (CaF_2_) had several advantages in that it reduces the dissolved silicon content of weld metal, prevents the deleterious effect of silicon on hot cracking, and lowers the melting range of the flux. Moreover, fluorine gases escaped from the weld pool interact with outer arc electrons leading to constrict arc [19]. The presence of fluorine in arc welding reduces the anode spot and tends to increase the energy density of the heat source and electromagnetic force in the weld pool. As a result, relatively narrow and deep weld morphology is formed [20,21]. Nineteen weld lines were executed and three samples were cut for the tensile test from each of the nineteen combinations.

### 2.9. Mathematical Modelling

For the first step of work, we developed a mathematical model where the UTS is written in terms of these selected oxides’ percentages. The technic used to get a mathematical model will be presented later in this paper. In the second step, the optimal combination that maximizes UTS is determined.

Finally, based on the optimal formulation, ATIG and TIG weld lines have been carried out on a single plate.

## 3. Results and Discussion

### 3.1. Tensile Test

#### 3.1.1. Selection of Candidate Oxides

Thirteen oxides were tested. The weld line was executed on the plain plate using welding parameters cited in Table 2. It can be seen in Table 3 that the highest value of UTS is 565 MPa, which was obtained for the sample welded with ZrO_2_ flux, followed by the sample welded with Cr_2_O_3_ flux with 559 MPa and, then by the sample welded with TiO_2_ flux with 542 MPa. Therefore, the selected oxides were Cr_2_O_3_, TiO_2_, and ZrO_2_.

#### 3.1.2. Mixture Design Combinations and UTS Response Values

Based on the simplex lattice degree four design, nineteen combinations have been prepared. As shown in Table 4, for each combination, the three selected oxides Cr_2_O_3_, TiO_2_, and ZrO_2_ vary and 25% of (8% SiO_2_ + 17% CaF_2_) is kept fixed.

Table 5 shows the Tensile test (UTS) results as well as the standards deviation (σ). The value of σ is less than 21 MPa for UTS results, which indicates that the disparities are acceptable.

#### 3.1.3. Mathematical Model

In this study, the three selected oxides (Cr_2_O_3_, TiO_2_, ZrO_2_) percentages vary for the combined oxide with a fixed 25% of (SiO_2_+ CaF_2_) oxide to form the pseudo-ternary ATIG are used. The UTS is then expressed as a function of the percentages of the three oxides as follows:

UTS = f (X1, X2, X3, X4). X1, X2, X3 denote, respectively, the proportions (in terms of percentages) of the three oxides, whereas the proportion of the fourth oxide is set to be X4 = 25%.

The chosen mathematical model that describes the effect of these percentages on the UTS is the coupling of second-order model as proposed in [22] with conventional linear regression as in [23]. This model includes three components: (i) the linear effect of the proportions, (ii) their quadratic effects, and (iii) the interactions between those proportions. The general form of the mathematical model is given below in expression 1:

UTS (predicted) = (α1)*X(1) + (α2)*X(2) + (α3)*X(3) + (α4)*X(1)*X(1) + (α5)*X(2)*X(2) + (α6)*X(3)*X(3) + (α7)*X(1)*X(2) + (α8)*X(1)*X(3) + (α9)*X(2)*X(3) + (α10)*X(4)(1)

With a fixed X(4) = 25%.

#### 3.1.4. Optimization Process Details

The first step objective was to find the optimal parameters that minimize the quadratic error between the measured UTS and the UTS provided by the model. Thus, the modeling problem is converted into an optimization problem having the model coefficients as decision variables and the quadratic error as the objective function to be minimized. Since this problem may present many irregularities such as non-convexity of the criterion and many feasibility constraints, we used a metaheuristic technique called particle swarm optimization (PSO) to solve this problem. In PSO, a set of candidate solutions (coefficients of the model) are initialized randomly within the search-space limits and then “flown” progressively toward a sub-optimal solution while combining three components: (i) follow their current velocities, (ii) go back to their best positions visited so far, and (iii) go to the position of the best neighbor. Each particle in the group (called swarm) is assigned four vectors: the position including the model parameters, a velocity, the best personal position, and the global best position. The move equations of each particle at the kth iteration of the optimization process are provided below [24,25]:

(2) $$\;V_{k+1}^{i}=w_{k}V_{k}^{i}+c_{1}r_{1}\left(P^{i}-\alpha^{i}\right)+c_{2}r_{2}\;\left(G^{i}-\alpha^{i}\right)\;$$

(3) $$\;\;\;\;\;\;\;\;\;\;\;\;\;\;\;\;\;\;\;\;\;\;\;\alpha_{k+1}^{i}=\alpha_{k}^{i}+V_{k+1}^{i}$$

(4) $$\;\;\;\;\;\;\;\;\;\;\;\;\;\;\;\;\;\;\;\;\;\;\;\;w_{k}=w_{max}-\frac{w_{max}-w_{min}}{k_{\max}}\times k$$

As provided in many papers such as [24,25], the inertia weight decreases linearly from 0.9 to 0.4. *c*_1_ = *c*_2_ = 0.75 are the cognitive and social factors. r_1_ and r_2_ are two random numbers generated randomly between 0 and 1. The model parameters’ search limits are set, respectively, to −1 and +1 for the overall model stability [25]. The optimization process is stopped after a certain number of iterations (k_max_ = 5000). After this global search step, a local search is conducted to find better solutions around the global sub-optimal solution yielded by the PSO algorithm. After running the optimization process many times, the selected UTS model as expressed by X1, X2, and X3 is given below:

(5) $$\begin{array}{cc} \mathrm{UTS}\;\left(\mathrm{predicted}\right) & \\ & =-0.7940\left({\%\mathrm{Cr}}_{2}O_{3}\right)+0.1706\left({\%\mathrm{TiO}}_{2}\right)+0.3442\left({\%\mathrm{ZrO}}_{2}\right)\\ & +0.1094\left({\%\mathrm{Cr}}_{2}O_{3}\right){\;}^{2}+0.0944\left({\%\mathrm{TiO}}_{2}\right){\;}^{2}+0.0959\left({\%\mathrm{ZrO}}_{2}\right){\;}^{2}\\ & +0.2032\left({\%\mathrm{Cr}}_{2}O_{3}\right)\left({\%\mathrm{TiO}}_{2}\right)+0.2173\left({\%\mathrm{Cr}}_{2}O_{3}\right)\left({\%\mathrm{ZrO}}_{2}\right)\\ & +0.2047\left({\%\mathrm{TiO}}_{2}\right)\left({\%\mathrm{ZrO}}_{2}\right)+0.4231 \end{array}$$

Three performance metrics are used to evaluate the effectiveness of the developed models as follows [25]:

Mean Absolute Percentage Error (MAPE (%)):

(6) $$\mathrm{MAPE}=\frac{100}{N_{2}}\overset{N_{2}}{\underset{t=1}{\sum }}\frac{\left|UTS\left(t\right)-UTSpredicted\left(t\right)\right|}{\;UTS\;mean}$$

- Coefficient of determination (R^2^ (%))
  (7) $$R^{2}=100\times \left(1-\frac{\frac{1}{N_{2}}\sum_{t=1}^{N_{2}}{\left(UTS\left(t\right)-UTSpredicted\left(t\right)\right)}^{2}}{\frac{1}{N_{2}}\sum_{t=1}^{N_{2}}{\left(UTS\left(t\right)-UTS\;mean\right)}^{2}}\right)$$
- Root Mean Square Error (RMSE (MPa))
  (8) $$\mathrm{RMSE}=\sqrt{\frac{1}{N_{2}}\;\overset{N_{2}}{\underset{t=1}{\sum }}{\left(UTS\left(t\right)-UTSpredicted\left(t\right)\right)}^{2}\;}\;$$
  With N_2_ = 19 (number of measurements)

The accuracy of this model as measured by the above cited measurement indicators are found to be as follows: MAPE = 0.7952%, R^2^ = 72.04%, and RMSE = 5.5955 MPa.

The second step of the optimization process consists of finding the optimal percentages of the three oxides that provide the maximum UTS. To achieve this goal, a constrained optimization algorithm is used. This algorithm has provided the following optimal combination shown in Table 6. The predicted value is UTS = 588 MPa as shown in Table 7.

#### 3.1.5. Experimental Validation

The validation test was the last step in the experimental process. A confirmation test was performed according to the optimum flux composition. Table 8 shows that UTS for ATIG weld reached 600 MPa, which is higher than that of conventional TIG welding (571 MPa). Moreover, the UTS of ATIG weld is greater than the expected value calculated by the mathematical model (588 MPa). The UTS of the optimal flux (ATIG) weld (600 MPa) is close to that of base metal (624 MPa) shown in Table 9. The ATIG welding can reduce the heat input per unit length in welds and the residual stress of the weld can be reduced [26,27]. On the other hand, the increase of UTS about 29 MPA in favor of ATIG comparatively to conventional TIG weld can be attributed to the increase of the retained ferrite volume proportions ATIG weld. Moreover, high heat input in TIG weld exhibits a coarse ferrite distribution and lower strength than the lower heat input in ATIG weld, which has a relatively fine ferrite distribution [27]. The value of standard deviation (σ) is less than 5 MPa.

#### 3.1.6. Tensile Break Zone Investigation

**Figure 6:**
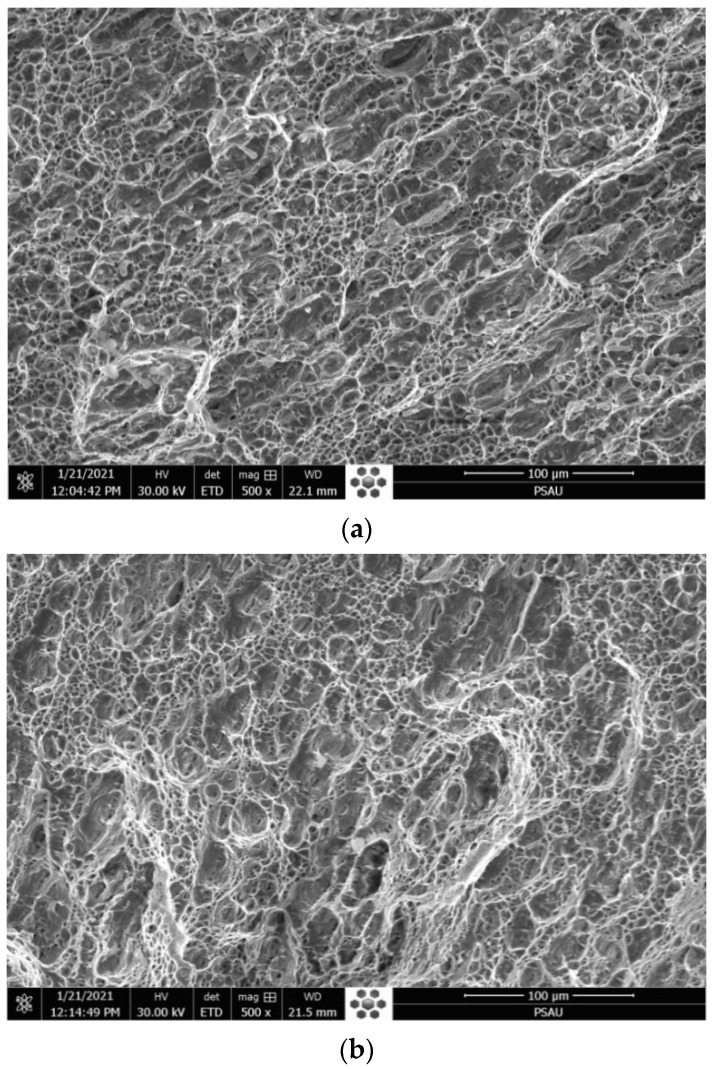
Fractograph of austenitic stainless steel 316L tensile test for TIG (**a**) and ATIG (**b**) welds (500×).

Micrographs of the break zone in the tensile test were conducted with SEM. The images show the same profile with the formation of multiple dimples, which demonstrates that the fracture is in the ductile mode for both cases of TIG and ATIG weld as shown in Figure 6a,b, respectively.

**Figure 7:**
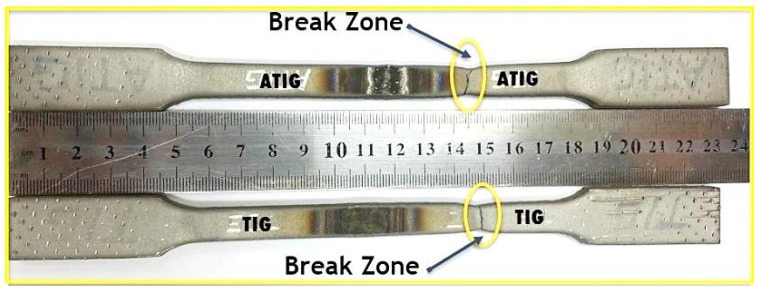
Failure zone in tensile test for ATIG and TIG welds.

We notice that the fracture location for both welds occurs in base metal as shown in Figure 7. The results show the elongation percentages very close to 22.63% for ATIG specimen against 20.52% for TIG weld.

### 3.2. Weld Bead Morphology

The values obtained for depth D and D/W ratio of the optimal flux ATIG weld are listed in Table 10. The results show that the depth of optimized flux is greater than TIG weld by two times. The ratio D/W was improved by 3.13 times. The weld of optimal flux is a full-penetrated weld (6.8 mm). The D/W value obtained for ATIG weld is 0.72.

**Figure 8:**
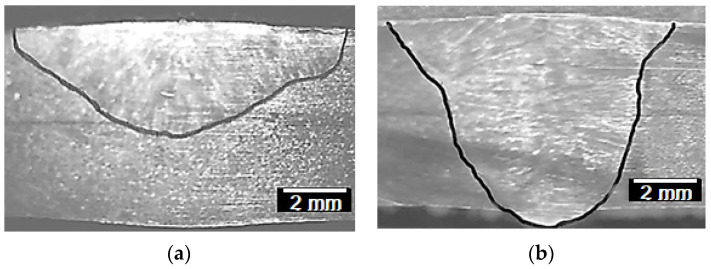
Morphology of TIG (**a**) and ATIG (**b**) welds.

This can be explained by the fact that Arc weld constriction and reversed Marangoni convection in ATIG occur when:

- Fluorine from flux migrates to the arc weld and contributes to enhancing the energy density according to the constriction arc mechanism explained before, as mentioned in several works [19,28].
- Oxygen liberated from oxides as surfactant element affects the surface tension of the molten metal resulting in a centripetal movement, the metal moves from the edges to the center as cited in related works [29,30]. A full penetration weld is performed in a single pass without edge preparation or the use of filler metal, which meets the needs of industries as shown in Figure 8b.

However, in TIG, Marangoni convection occurs. A molten metal moves from the center of the weld pool to the edges as pure metal leading to a wide and shallow weld bead as shown in Figure 8a.

### 3.3. Microstructural Assessment

**Figure 9:**
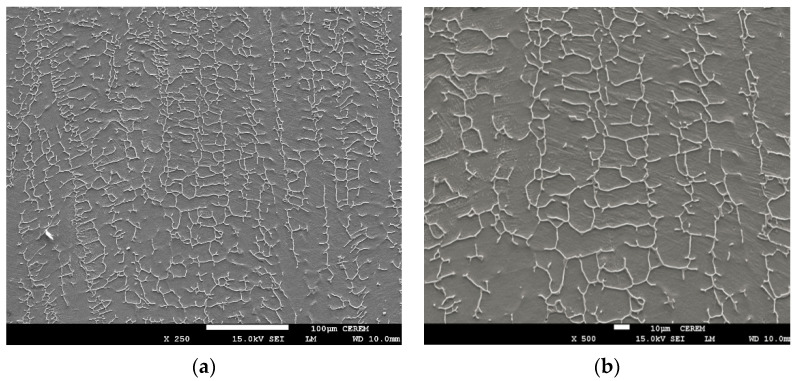
SEM micrograph in TIG fusion zone of 316L (**a**) (250×), (**b**) (500×).

**Figure 10:**
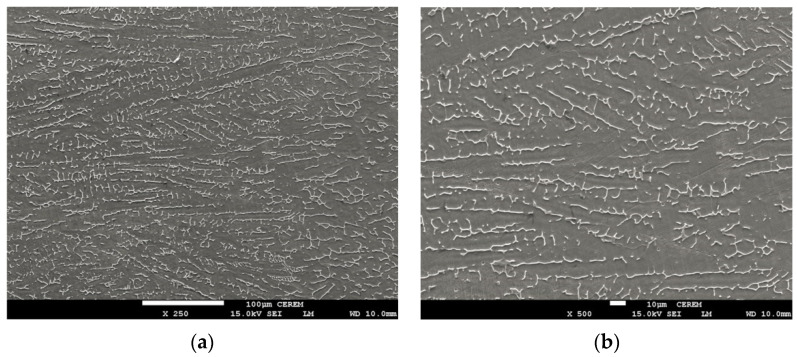
SEM Micrograph in ATIG fusion zone of 316L (**a**) (250×), (**b**) (500×).

The differences in microstructure between TIG weld bead and ATIG weld are shown in Figure 9a,b and Figure 10a,b respectively. In both cases, the ferrite morphology has a discontinuous skeletal network of delta ferrite (δ) structures in a predominant austenite matrix. Alloy 316L with Creq/Nieq that is 1.67 solidifies in ferritic austenitic mode [31]. The delta ferrite is located mainly at the dendrite axes. The residual primary ferrite results from incomplete δ → γ transformation during solidification.

The delta ferrite (δ) in the TIG weld zone is coarser than that of the ATIG weld as shown in Figure 9b and Figure 10b, respectively. This difference in delta ferrite (δ) size is related to the heat provided by the weld bead, which is lesser in the case of ATIG weld.

**Figure 11:**
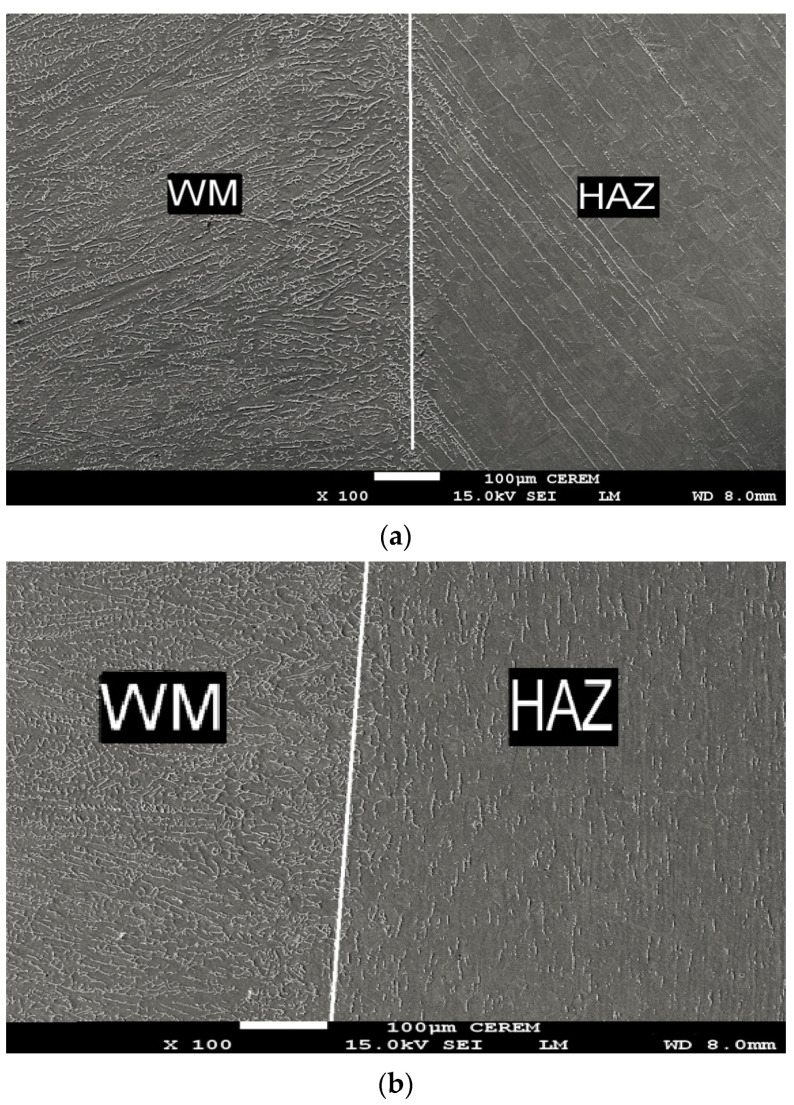
SEM Micrograph in transition zone (WZ/HAZ) in ATIG weld (**a**) and TIG weld (**b**) (100×).

In ATIG and TIG weld zone, the ferrite is the result of the incomplete primary delta ferrite- austenite transformation. The proportions of delta ferrite diminish from weld zone to base metal is as shown in Figure 11a,b.

In the ATIG heat-affected zone, austenitic grains crossed by parallel elongated inclined stringers of delta ferrite as shown in Figure 11a. In TIG heat-affected zone, the globular delta ferrite is randomly distributed and in the form of small slats of reduced size in the same rolling direction in a matrix of austenite as shown in Figure 11b.

**Figure 12:**
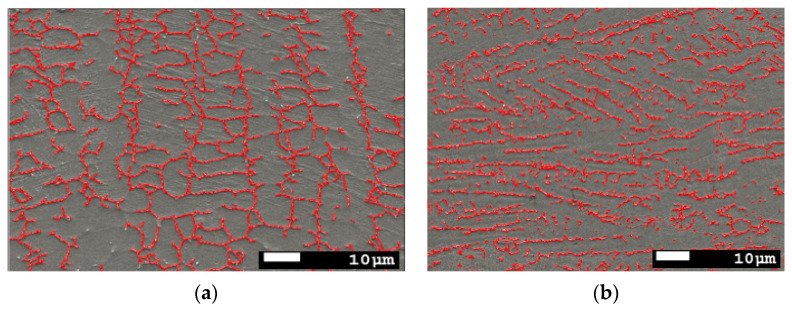
Ferrite proportions measurements of 316L, (**a**) TIG Weld Zone and (**b**) ATIG Weld Zone (SEM. magnification 500×).

Figure 12a,b represent the measurements of ferrite volume proportions in the austenite matrix. The advisable proper amount of δ-ferrite in austenitic stainless steel welds is less than 10% volume to ensure a better ductility, toughness, and corrosion resistance, and no less than 5% to avoid solidification cracking [32].

The results reported in Table 11 show the measurements taken in seven different locations in the weld zone and the mean value of ferrite volume. The proportions are up to 5.56% in the conventional TIG process and 8.63% for ATIG weld.

The higher retained ferrite may be attributed to the rate of cooling of the welds. Elements from optimal flux can constrict arc weld. The constriction of the arc will increase the temperature at the anode due to the increase in current density and arc voltage as reported in many studies [33,34].

The ATIG welding with optimal flux increased the energy density of the heat source that leads to low heat input. However, the arc heat of TIG welding without flux has a lower energy density; therefore, high heat input is provided to the workpiece. High heat input resulted in a slow cooling rate and a further transformation from ferrite phase to the austenite phase. Consequently, a ferrite volume proportion is reduced (5.56%). The ATIG welding was associated with a low heat input resulting in higher ferrite content (8.63%), which is in good agreement with several works [35,36]. On the other hand, the high heat input leads to coarse substructure during solidification in the case of TIG weld as shown in Figure 9a,b, which results in a more widely spaced ferrite network. But in the case of ATIG weld, heat provided is low; a fast cooling rate occurs and consequently a finer skeletal ferrite is formed as shown in Figure 10a,b [37].

### 3.4. Hardness Test

The hardness values are shown in Table 12. It is visible that the hardness of ATIG weld and TIG weld in both FZ and HAZ are very close. The delta-ferrite volume proportions in the weld metals are in both TIG and ATIG welds increased, and have a beneficial effect in increasing the hardness of as received 316L stainless steel welds (160 HV).

In ATIG weld, the property of hardness is not affected by the optimal flux used. On the other hand, the standard deviation is less than 9 HV, which attests to the small disparities in the obtained hardness values of the maximum and minimum. This result indicates good hardness homogeneities in the joints.

### 3.5. Impact Test

The impact tests were carried out on the fusion zone in ATIG and TIG welds. The experimental values obtained for the impact tests are shown in Table 13. The energy absorbed in the fusion zone in the case of ATIG weld (267 J/cm^2^) is slightly higher than that of TIG weld (256 J/cm^2^) by 11 J/cm^2^. The standard deviation is less than 15 J/cm^2^.

**Figure 13:**
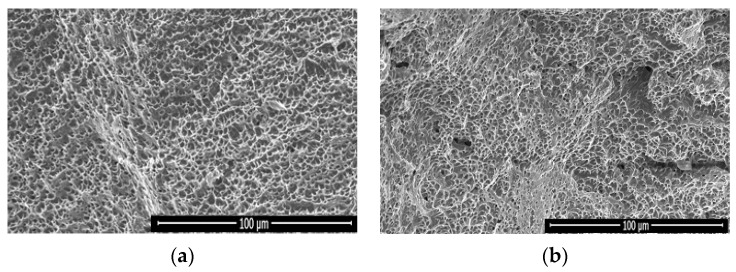
Fractograph of Austenitic stainless steel 316L impact Charpy “V” notch for (**a**) TIG Welded Zone (500×) and (**b**) ATIG Welded Zone (500×).

Figure 12 represents the fractographs of the impact Charpy “V” notch test. The images show the formation of multiple dimples, which demonstrate that the fracture is a ductile mode in both cases of TIG and ATIG as shown in Figure 13a,b. However, in ATIG weld the dimples are finer with the presence of voids, which can explain the slightly high value of absorbed energy comparatively to TIG weld. The ductile fracture mode leads to good resistance to sudden impact loads. Multiple dimples attest for high impact energy withstand as reported by several authors [38,39].

**Figure 14:**
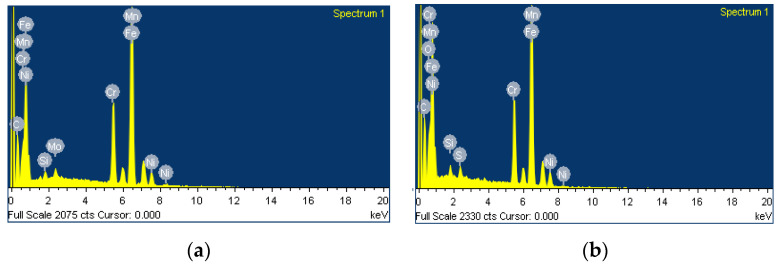
EDS/SEM spectrum analysis of fracture face of ATIG welded zone (**a**) and TIG welded zone (**b**).

The results of EDS/SEM analysis in Figure 14a and Table 14 are shown in the case of ATIG weld, with the same level of silicon as in base metal. There are no trace of unwanted elements in the case of ATIG welds. These results can explain the good resistance to impact test.

The results of EDS/SEM analysis in Figure 14b and in Table 14 show the presence of oxygen in TIG weld, which is probably ascribed to insufficient protection of the weld pool that affects mechanical properties, particularly the toughness. The presence of sulfur leads to the formation of low-melting eutectics with iron, chromium, and nickel, which can alter the mechanical properties of TIG weld beads [40]. The obtained results with EDS/SEM related to TIG weld may explain the decrease in resistance to impact test comparatively to the ATIG weld.

## 4. Conclusions

In the present work, ATIG weld has been investigated and compared to conventional TIG weld. The starting point of this work is elaborating the optimal flux to maximize the UTS ATIG weld. The mixing design of the experiment combined to the particle swarm optimization (PSO) method is used to minimize the number of trials, which reduces the cost of materials as well as time. Based on the obtained results in this investigation, the following conclusions can be drawn:

- Mixing design of the experiment combined to the particle swarm optimization (PSO) method is among the novelties of this work. Optimal flux was composed by 32% Cr_2_O_3_, 43% ZrO_2_, 8% SiO_2_, and 17% CaF_2_. The optimal flux raised from this method improves the mechanical properties in comparison to conventional TIG weld bead. Transversal tensile testing of the produced ATIG weld has a UTS value (600 MPa) close to parent metal (624 MPa). On the other hand, the UTS of TIG weld fell to 571 MPa. ATIG welding is more resistant than conventional TIG welding to sudden impact loads. The ATIG weld hardness results were close to those of conventional TIG weld.
- The ATIG depth weld bead reached 6.80 mm and the weld aspect ratio increased 3.13. The ATIG depth weld was increased by two times in comparison to the conventional TIG weld bead. The fully penetrated ATIG weld is ascribed to two mechanisms cited earlier. The reversal Marangoni mechanism owing to oxygen liberated from flux and the constriction of ATIG arc weld related to the migration of fluorine from the flux to the arc.
- The microstructure of welds in both welds is composed of matrix austenite interspersed by skeleton δ-ferrite. The ferrite volume proportions in ATIG weld around 8.63% and decreased to 5.56% in conventional TIG welding. The δ-ferrite in TIG weld is coarser comparatively to that of ATIG δ-ferrite.

## Figures and Tables

**Table 1:** Chemical composition of 316L stainless steel.

Elements	C	Mn	Si	P	S	Cr	Ni	Mo	N	Cu	Fe
Weight %	0.026	1.47	0.42	0.034	0.0016	16.60	10.08	2.14	0.044	0.50	Balance

**Table 2:** Welding parameters.

Parameters	Range
Welding speed	150 mm/min
Welding current	180 A
Arc length	2 mm
Electrode tip angle	45°
Shielding gas on the workpiece	Argon with flow rate 10 L/min
Shielding gas on the backside	Argon with flow rate 5 L/min
Polarity	DCEN

**Table 3:** UTS (MPa) for different ATIG mono-oxide welds.

Oxide	SiO_2_	TiO_2_	Fe_2_O_3_	MnO_2_	Cr_2_O_3_	ZrO_2_	CaO	Mn_2_O_3_	V_2_O_5_	MoO_3_	SrO	Co_2_O_3_	MgO
UTS	529	542	538	539	559	565	536	534	481	491	338	506	523

**Table 4:** Different pseudo-ternary combinations with the three selected oxides.

Exp. No.	Cr_2_O_3_ [Weight %]	TiO_2_ [Weight %]	ZrO_2_ [Weight %]	SiO_2_ + CaF_2_ [Weight %]
1	56.25	18.75	0.00	25
2	56.25	0.00	18.75	25
3	37.50	18.75	18.75	25
4	37.50	0.00	37.50	25
5	37.50	37.50	0.00	25
6	18.75	56.25	0.00	25
7	18.75	37.50	18.75	25
8	18.75	18.75	37.50	25
9	0.00	56.25	18.75	25
10	0.00	37.50	37.50	25
11	0.00	18.75	56.25	25
12	25.00	25.00	25.00	25
13	50.00	12.50	12.50	25
14	12.50	50.00	12.50	25
15	12.50	12.50	50.00	25
16	75.00	0.00	0.00	25
17	0.00	75.00	0.00	25
18	0.00	0.00	75.00	25
19	18.75	0.00	56.25	25

**Table 5:** UTS of a set of experiments with the different combinations and the related standard deviation σ.

Exp. No.	Number of Tests	Max. UTS [MPa]	Min. UTS [MPa]	Average UTS [MPa]	σ Standard Deviation
1	3	577	552	564	12.58
2	3	592	587	590	2.51
3	3	595	575	578	16.16
4	3	590	557	579	19.05
5	3	570	538	551	16.82
6	3	577	541	558	18.14
7	3	594	560	582	19.34
8	3	590	556	579	19.62
9	3	585	557	575	15.88
10	3	600	563	587	20.55
11	3	594	561	581	17.78
12	3	594	558	582	20.78
13	3	592	558	579	18.35
14	3	596	560	582	19.50
15	3	593	562	582	17.61
16	3	570	558	563	6.02
17	3	555	542	550	6.80
18	3	585	565	580	11.54
19	3	593	591	592	1.15

**Table 6:** The optimal composition of flux.

Variables	Cr_2_O_3_	TiO_2_	ZrO_2_	SiO_2_	CaF_2_
Single Percentages	32%	0%	43%	8%	17%
Combined Percentages	75% (Cr_2_O_3_ + ZrO_2_)			25% (SiO_2_ + CaF_2_)	

**Table 7:** Predicted responses UTS of weld executed with optimal flux.

Response	Predicted Response (MPa)
UTS	588.27

**Table 8:** Results of UTS (MPa) and standard deviation of TIG and ATIG.

Sample	Number of Tests	UTS Max.	UTS Min.	UTS Mean	Standards Deviation (σ)
TIG	4	578	568	571	4.57
ATIG	4	602	596	600	2.1

**Table 9:** UTS comparison between mathematical model, ATIG, TIG, and base metal.

Sample	UTS (MPa)
Expected value by mathematical model	588
Optimal combination (ATIG)	600
TIG	571
Base metal (SS316L)	624

**Table 10:** Weldment bead profiles data of TIG and ATIG.

TIG			ATIG		
D (mm)	W (mm)	D/W	D (mm)	W (mm)	D/W
3.26	14.44	0.23	6.80	9.5	0.72

**Table 11:** Ferrite volume fraction measurement.

Sample	Number of Measurements	Max. δ-Ferrite %	Min. δ-Ferrite %	Mean δ-Ferrite %	Standards Deviation σ
TIG	7	6.2	4.8	5.56	0.35
ATIG	7	9	8.1	8.63	0.31

**Table 12:** Hardness values and standards deviation of TIG and ATIG.

Sample	Number of Tests	HV Max.	HV Min.	HV Mean	Standards Deviation σ
ATIG FZ	8	188	176	183	4.93
TIG FZ	8	199	175	185	8.98
ATIG HAZ	8	177	167	171	3.41
TIG HAZ	8	180	164	172	5.03

**Table 13:** Energy absorbed (J/cm^2^) and standard deviation of TIG and ATIG at fusion zone.

Sample	Number of Tests	Absorbed Energy Min.	Absorbed Energy Max.	Absorbed Energy Mean	Standards Deviation (σ)
TIG	3	241	269	256	14.05
ATIG	3	254	281	267	13.58

**Table 14:** Elements present in fracture face of ATIG welded zone and TIG welded zone.

Sample		C %	Si %	Cr %	Mn %	Fe %	Ni %	Mo %	O %	S %
ATIG weld	Weight % Atomic %	16.26 47.29	0.43 0.54	14.80 9.94	1.75 1.11	58.10 36.34	7.09 4.22	1.57 0.54		
TIG weld	Weight % Atomic %	17.898 47.49	0.68 0.77	14.86 9.11	1.39 0.81	54.67 31.21	6.73 3.65		3.20 6.38	0.58 0.58

## Data Availability

The data used to support the findings of this study are included within the article.

## References

[1] Tathgir S., Bhattacharya A. (2016). Activated-TIG Welding of Different Steels: Influence of Various Flux and Shielding Gas. Mater. Manuf. Process..

[2] Klobčar D., Tušek J., Bizjak M., Simončič S., Lešer V. (2016). Active flux tungsten inert gas welding of austenitic stainless steel AISI 304. Metalurgija.

[3] Modenesi P.J., Neto C.P., Apolinario E.R., Dias B.K. (2015). Effect of flux density and the presence of additives in ATIG welding of austenitic stainless steel. Weld. Int..

[4] Kumar R., Sundara Bharathi S.R. (2015). A Review Study on A-TIG Welding of 316(L) Austenitic Stainless Steel. Int. J. Emerg. Trends Sci. Technol. (IJETS).

[5] Howse D.S., Lucas W. (2000). An investigation in to arc construction by active flux for TIG welding. Sci. Technol. Weld. Join..

[6] Kurtulmuş M. (2020). Activated flux TIG welding of austenitic stainless steels. Emerg. Mater. Res..

[7] Sire S., Marya S. (2002). On the development of a new flux bounded TIG process (FBTIG) to enhance weld penetrations in aluminium, 5086. Int. J. Form. Process..

[8] Jayakrishnan S., Chakravarthy P., Muhammed Rijas A. (2017). Effect of Flux Gap and Particle Size on the Depth of Penetration in FBTIG Welding of Aluminium. Trans. Indian Inst. Met..

[9] Ambekar S.D., Wadhokar S.R. (2015). Parametric Optimization of Gas metal arc welding process by using Taguchi method on stainless steel AISI 410. Int. J. Res. Mod. Eng. Emerg. Technol..

[10] Srirangan A.K., Paulraj S. (2016). Multi-response optimization of process parameters for TIG welding of Incoloy 800HT by Taguchi grey relational analysis. Eng. Sci. Technol. Int. J..

[11] Ramadan N., Boghdadi A. (2020). Parametric Optimization of TIG Welding Influence On Tensile Strength of Dissimilar Metals SS-304 And Low Carbon Steel by Using Taguchi Approach. Am. J. Eng. Res..

[12] Chaudhari V., Bodkhe V., Deokate S., Mali B., Mahale R. (2019). Parametric optimization of TIG welding on SS 304 and MS using Taguchi approach. Int. Res. J. Eng. Technol..

[13] Albaijan I., Hedhibi A.C., Touileb K., Djoudjou R., Ouis A., Alrobei H. (2020). Effect of Binary Oxide Flux on Weld Shape, Mechanical Properties and Corrosion Resistance of 2205 Duplex Stainless Steel Welds. Adv. Mater. Sci. Eng..

[14] Touileb K., Ouis A., Djoudjou R., Hedhibi A.C., Alrobei H., Albaijan I., Alzahrani B., Sherif M.E., Abdo H.S. (2020). Effects of ATIG Welding on Weld Shape, Mechanical Properties, and Corrosion Resistance of 430 Ferritic Stainless Steel Alloy. Metals.

[15] Ran L., Manshu D., Hongming G. (2021). Prediction of Bead Geometry with Changing Welding Speed Using Artificial Neural Network. Materials.

[16] Kshirsagar R., Jones S., Lawrence J., Tabor J. (2019). Prediction of Bead Geometry Using a Two-Stage SVM–ANN Algorithm for Automated Tungsten Inert Gas (TIG) Welds. J. Manuf. Mater. Process..

[17] Las-Casas M.S., De Ávila T.L.D., Bracarense A.Q., Lima E.J. (2018). Weld parameter prediction using artificial neural network: FN and geometric parameter prediction of austenitic stainless steel welds. J. Braz. Soc. Mech. Sci. Eng..

[18] Tseng K.H., Chen K.L. (2012). Comparisons between TiO_2_- and SiO_2_-Flux Assisted TIG Welding Processes. J. Nanosci. Nanotechnol..

[19] Leconte S., Paillard P., Chapelle P., Henrion G., Saindrenan J. (2013). Effects of flux containing fluorides on TIG welding process. Sci. Technol. Weld. Join..

[20] Neethu N., Togita R.G., Neelima P., Chakravarthy P., Narayana M.S.V.S., Nair M.T. (2019). Effect of Nature of Flux and Flux Gap on the Depth-to-Width Ratio in Flux-Bounded TIG Welding of AA6061: Experiments and Numerical Simulations. Trans. Indian Inst. Met..

[21] Babbar A., Kumar A., Jain V., Gupta D. (2019). Enhancement of Activated Tungsten Inert Gas (A-TIG) Welding Using Multi-component TiO2-SiO2-Al2O3 Hybrid Flux. Measurement.

[22] Ahmed A.N., Noor C.W.M., Allawi M.F., El-Shafie A. (2018). RBF-NN-based model for prediction of weld bead geometry in Shielded Metal Arc Welding (SMAW). Neural Comput. Appl..

[23] Kumar R., Saurav S.K. (2015). Modeling of TIG welding process by regression analysis and neural network technique. Int. J. Mech. Eng. Technol. (IJMET).

[24] Kshirsagar R., Jones S., Lawrence J., Tabor J. (2020). Optimization of TIG Welding Parameters Using a Hybrid Nelder Mead-Evolutionary Algorithms Method. J. Manuf. Mater. Process..

[25] Boubaker S., Kamel S., Kolsi L., Kahouli O. (2020). Forecasting of One-Day-Ahead Global Horizontal Irradiation Using Block-Oriented Models Combined with a Swarm Intelligence Approach. Nat. Resour. Res..

[26] Patel N.P., Badheka V.J., Vora J.J., Upadhyay G.H. (2019). Effect of Oxide Fluxes in Activated TIG Welding of Stainless Steel 316LN to Low Activation Ferritic/Martensitic Steel (LAFM) Dissimilar Combination. Trans. Indian Inst. Met..

[27] Goodwin G.M., Cole N.C., Slaughtera D.G.M. (1972). Study of Ferrite Morphology in Austenitic Stainless Steel Weldments. Weld. Res. Suppl..

[28] Ming L.Q., Hong W.X., Da Z.Z., Jun W. (2007). Effect of activating flux on arc shape and arc voltage in tungsten inert gas welding. Trans. Nonferrous Met. Soc. China.

[29] Kulkarni A., Dwivedi D.K., Vasudevan M. (2018). Study of Mechanism, Microstructure and Mechanical Properties of Activated Flux TIG Welded P91 Steel-P22 Steel Dissimilar Metal Joint. Mater. Sci. Eng. A.

[30] Vora J.J., Badheka V.J. (2015). Experimental Investigation on Mechanism and Weld Morphology of Activated TIG Welded Bead-on-plate Weldments of Reduced Activation Ferritic/martensitic Steel Using Oxide Fluxes. J. Manuf. Process..

[31] Kujanpaa V.P., Suutala V.P., Takalo N.J., Moisio T.J.I. (1980). Solidification Cracking—Estimation of the Susceptibility of Austenitic and Austenitic-Ferritic Stainless Steel Welds. Met. Constr..

[32] Kou S. (2003). Welding Metallurgy.

[33] Dixit P., Suketu J. (2021). Techniques to weld similar and dissimilar materials by ATIG welding—An overview. Mater. Manuf. Process..

[34] Roy S., Samaddar S., Uddin M.N., Hoque A., Mishra S., Das S. (2017). Effect of Activating Flux on Penetration in ATIG Welding of 316 Stainless Steel. Indian Weld. J..

[35] Vasudevan M. (2017). Effect of A-TIG Welding Process on the Weld Attributes of Type 304LN and 316LN Stainless Steels. J. Mater. Eng. Perform..

[36] Suman S., Santanu D. (2020). Effect of Polarity and Oxide Fluxes on Weld-bead Geometry in Activated Tungsten Inert Gas (A-TIG) Welding. J. Weld. Join..

[37] Lippold J.C., Savage W.F. (1980). Solidification of Austenitic Stainless Steel Weldments: Part 2-The Effect of Alloy Composition on Ferrite Morphology. Weld. J..

[38] Jebaraj A.V., Kumar T.S., Manikandan M. (2018). Investigation of Structure Property Relationship of the Dissimilar Weld Between Austenitic Stainless Steel 316L and Duplex Stainless Steel 2205. Trans. Indian Inst. Met..

[39] Charles J. (1995). Composition and properties of duplex stainless steels. Weld. World.

[40] Harish K.D., Somi R.A. (2013). Study of Mechanical Behavior in Austenitic Stainless Steel 316 LN Welded Joints. Int. J. Mech. Eng. Rob. Res..

